# Retrospective Whole-Genome Sequencing and Molecular Epidemiological Analysis of Austrian WNV-2 Veterinary Cases (2008–2025)

**DOI:** 10.3390/pathogens15101061

**Published:** 2026-10-08

**Authors:** Michaela Stätter, Daniel Polzer, Katharina Reisp, Friedrich Schmoll, Anna Stöger, Sandra Revilla-Fernández

**Affiliations:** 1 Institute for Veterinary Disease Control, Austrian Agency for Health and Food Safety (AGES), 2340 Mödling, Austria; 2 Institute of Medical Microbiology and Hygiene, Austrian Agency for Health and Food Safety (AGES), 1090 Vienna, Austria

**Keywords:** West Nile virus, WNV-2, whole-genome sequencing, NGS, epidemiology, veterinary, molecular epidemiology

## Abstract

The West Nile virus of the lineage 2 (WNV-2) has become endemic in large parts of Europe; however, comprehensive whole-genome sequence datasets documenting long-term national circulation are still sparse. This study aimed for the retrospective genetic characterization of WNV-2 detected in animals in Austria between the first introduction in 2008 and 2025 using whole-genome sequencing. Phylogenetic analyses of the sequences allowed the viral diversity, lineage dynamics and molecular epidemiology to be investigated within a broader European context. Twenty-four Austrian WNV-2-positive samples originating from birds (22), one horse and one alpaca were subjected to in-house whole-genome amplification and Illumina sequencing. The generated genomes were analyzed using maximum-likelihood phylogenies and a European WNV-2 Nextstrain build comprising approximately 1000 publicly available whole-genome sequences. Whole-genome sequences were successfully recovered for all selected samples, indicating a reliable whole-genome amplification method for the chosen Austrian strains. The phylogenetic analysis identified three different WNV-2 subclades circulating in Austria (D2, E1 and E2) and revealed multiple periods of subclade co-circulation. Furthermore, Austria and Hungary were inferred as important geographic locations in the diversification and dissemination of WNV-2 in Europe. These findings demonstrate the dynamic circulation as well as the repeated introduction of WNV-2 subclades into Austria and highlight the value of whole-genome sequencing surveillance for monitoring lineage turnover and improving the understanding of WNV-2 evolution across Europe.

## 1. Introduction

West Nile virus (WNV) is a mosquito-borne virus belonging to the family *Flaviviridae*, with known zoonotic potential. It is maintained in nature in an enzootic transmission cycle between mosquitoes, as virus vectors, and birds, which serve as the main reservoir and amplifying host of the virus [1,2,3,4]. In contrast, humans and several mammalian species, including horses, are regarded as incidental or dead-end hosts [2,3,5,6]. Within Europe, the WNV lineage 2 (WNV-2) has become the dominant lineage in many affected regions and is commonly subdivided into two major phylogenetic clades, although no official nomenclature exists [3,7,8]: the central European clade (CEC, also termed WNV-2a or clade E) and the south-eastern European clade (SEEC, also termed WNV-2b or clade D). Various authors have proposed subsequent subclusters thereof, often reflecting the geographic origin or regional circulation patterns [6,7,8,9,10,11,12,13,14].

In 2008, six reported WNV-2 cases in five goshawks and one gyrfalcon marked the first WNV detections in Austria [15]. Prior to the Austrian cases, a goshawk WNV-2 case in Hungary from 2003 was described [4]. Ever since the first cases in 2008, the continued seasonal presence of WNV-2 in different hosts such as humans, birds (especially in birds of prey), horses and mosquitos in Austria has been well documented [5,16,17,18,19,20]. Since 2009, autochthonous human infections have been reported almost annually, although the case numbers have varied considerably between transmission seasons, with pronounced increases during the exceptional European outbreak year 2018 and again in 2024 [5,6,20].

Before the emergence of WNV-2 in Europe, this WNV lineage was deemed to be less pathogenic than WNV-1 [21]. On the contrary, WNV-2 has been shown to cause severe and sometimes fatal neuroinvasive disease in humans, horses and several avian species, thereby disproving the assumption that lineage 2 is of low pathogenicity [5,6,9,10,15,17,20,22]. In horses, infection may result in marked neurological signs and death or euthanasia in severe cases, highlighting the veterinary relevance of WNV-2 surveillance and prevention. Importantly, prophylactic vaccines are available for equids and constitute a key preventive measure in endemic and newly affected areas [2,20,23]. In birds, particularly raptors, WNV-2 infection has repeatedly been associated with increased susceptibility, neurological disease and mortality, making the virus a particular threat to small, valuable or conservation-relevant captive populations [1,24].

In many surveillance and outbreak investigations, the phylogenetic characterization of WNV-2 has traditionally relied on RT-PCR assays followed by Sanger sequencing of partial genomic regions, frequently including the NS5 gene [5,6,18]. However, with the recent advances in whole-genome sequencing (WGS), next-generation sequencing (NGS) has become a powerful tool for epidemiological investigation, as it offers a substantially higher phylogenetic resolution. Consequently, a more insightful reconstruction of viral entry and transmission routes, viral lineage determination, and a historical assessment of the circulating lineage distribution in detected cases can be conducted. Previous studies from European countries utilizing WGS-based phylogenies focusing on WNV-2 specifically showed that WNV-2 circulation is shaped by repeated dispersal events and geographic clustering, rather than by uniform linear spread alone [7,8,9,10,11,25,26]. Therefore, Austria’s unique central geographical location with respect to central, eastern and southern Europe makes an analysis of WNV-2 movement across borders highly relevant in understanding the transmission pathways within the European transmission network.

The aim of this study was to retrospectively characterize Austrian WNV-2 strains detected in animals collected between 2008 and 2025 using whole-genome sequencing. Phylogenetic analyses of the resulting sequences were done to investigate the viral diversity, lineage dynamics, and molecular epidemiology of WNV-2 in Austria within a broader European context, as well as to provide a public Austrian WNV-2 sequence dataset representative of our archived material since the first detection in birds in 2008.

## 2. Materials and Methods

### 2.1. Sample Selection

For the retrospective analysis, previously WNV-2-confirmed archived samples at the National Reference Laboratory for WNV in animals of the AGES Institute for Veterinary Disease Control in Mödling were selected, depending on the Cq-value (<28) of the samples determined by real-time RT-PCR and the total nucleic-/RNA-extract availability [27]. The selected samples adequately reflected the annual distribution of the detected cases by molecular-based methods, with more than 32% of all confirmed cases by molecular testing recorded during the 2008–2025 study period successfully sequenced. For four samples collected between 2008 and 2014, no Cq-value was available, as during this time, molecular diagnostics were performed through a two-step heminested RT-PCR [28]. The initial matrices included the pooled organs, brain tissue and spleen of birds, horses and one alpaca. The sample identifiers, matrices, host species, respective WNV real-time RT-PCR Cq-values and sampling dates are indicated in Table 1.

### 2.2. Whole-Genome Amplification Primer Set Design Process

**Figure 1:**
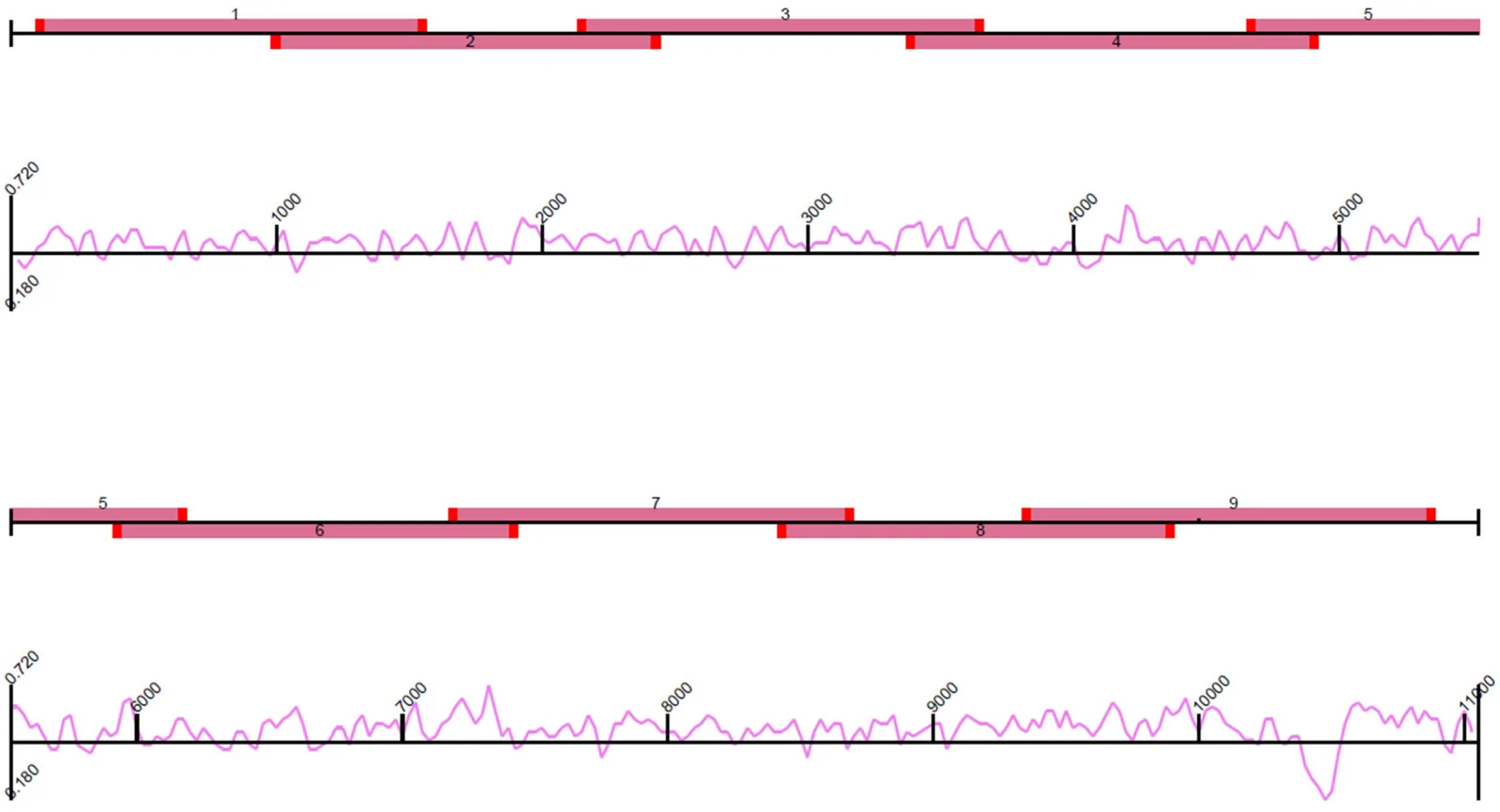
WNV-2 whole-genome amplicon (WGA) scheme generated using primalscheme [29]. Overlapping amplicons are shown as pink bars and primer-binding sites as red bars. The purple line represents the local GC content calculated across the reference genome using a 50-nucleotide sliding window.

Each sample was subjected to a tiled in-house one-step RT-PCR method for whole-genome amplification. The respective primer sets (PCR1 and PCR2, listed in Table 2, as well as illustrated in the primalscheme plot in Figure 1), containing a total of 9 primer pairs (indicated in Table 2), were designed with primalscheme (version 1.4.1) according to the steps outlined in the corresponding GitHub repository [29] (https://github.com/aresti/primalscheme (accessed on 31 July 2024)). For the creation of the two primer sets, the sequence EF429198.1 was chosen as the primary reference and the sequences MW383508.1, NC_001563.2, and OK239667.1 were used as secondary references by primalscheme. The input settings included a 1500 bp amplicon length, as well as a 100 bp overlap. The amplicon length was chosen according to the amplicon length limit conferred by the utilized one-step RT-PCR kit, the percent coverage of the WNV-2 whole genome and the possibility to use the obtained amplicons for both short- and long-read NGS approaches.

### 2.3. Whole-Genome Amplification and Short-Read Sequencing

For whole-genome amplification, the SuperScript™ III One-Step RT-PCR System Platinum™ Taq DNA Polymerase (Life Technologies, Carlsbad, CA, USA) was used according to the manufacturer’s guidelines for both PCRs (PCR1 and PCR2) with a 62 °C annealing temperature and a final concentration of each primer of 0.2 pmol/µL. The successful amplification of each PCR was confirmed via automated gel electrophoresis using the D5000 Screentape on the Agilent Tapestation (Agilent Technologies Inc., Santa Clara, CA, USA). Successfully amplified PCR reactions showcasing visible bands of the expected amplicon size were pooled equidistantly and sent to the AGES NGS-Core facility for paired-end short-read sequencing. The Illumina sequencing library was prepared using the NexteraXT kit and finally sequenced on a NextSeq2000 with 2 × 150 bp (Illumina, San Diego, CA, USA). Raw reads were trimmed with fastp (version 1.3.6). To identify the most appropriate reference genome for each sample, the sequencing reads were screened against a custom multi-FASTA database of representative WNV-2 genomes (corresponding accession numbers and metadata can be found in Supplementary Materials S2) using kma (version 1.4.15) [30]. The reference sequence with the highest template score and read coverage was selected. The reads were remapped to the selected reference genome and consensus sequences were generated using kma with standard settings for paired-end reads according to the developer’s recommendations. The threshold for calling a consensus base was set to 0.7. The obtained whole-genome sequences were uploaded intothe NCBI Genbank (accession numbers PZ698354-PZ698377).

### 2.4. Time-Annotated Phylogenetic Analysis of WNV-2 Whole-Genome Sequences Detected in Austria Between 2008 and 2025

The WNV-2 sequences obtained during this study were combined with the available published Austrian sequences on NCBI (see table in Supplementary Materials S3) and aligned with MAFFT (version 7.525). A maximum-likelihood phylogeny was inferred using IQ-TREE (version 2.3.6) with 1000 Ultrafast bootstrap replicates, and the best-fitting model was determined by the embedded ModelFinder Plus (MFP) algorithm. To check the dataset representativeness and obtain a time-annotated tree, the whole-genome sequences combined with the respective data were subjected to TreeTime (version 0.11.4) to estimate the nucleotide substitution rate and the corresponding R^2^ as well as possible outliers.

The resulting tree was then annotated with the respective clades determined in previous publications [8,14] via VSEARCH (version 2.30.3); the references used for the respective subclades are listed in Table 3. The final subclade assignment was determined by the obtained phylogeny.

The determined subclades are indicated in the created time-annotated Austrian phylogeny in the results.

### 2.5. Phylogeny and Epidemiological Analysis in the Broader European Context

**Figure 2:**
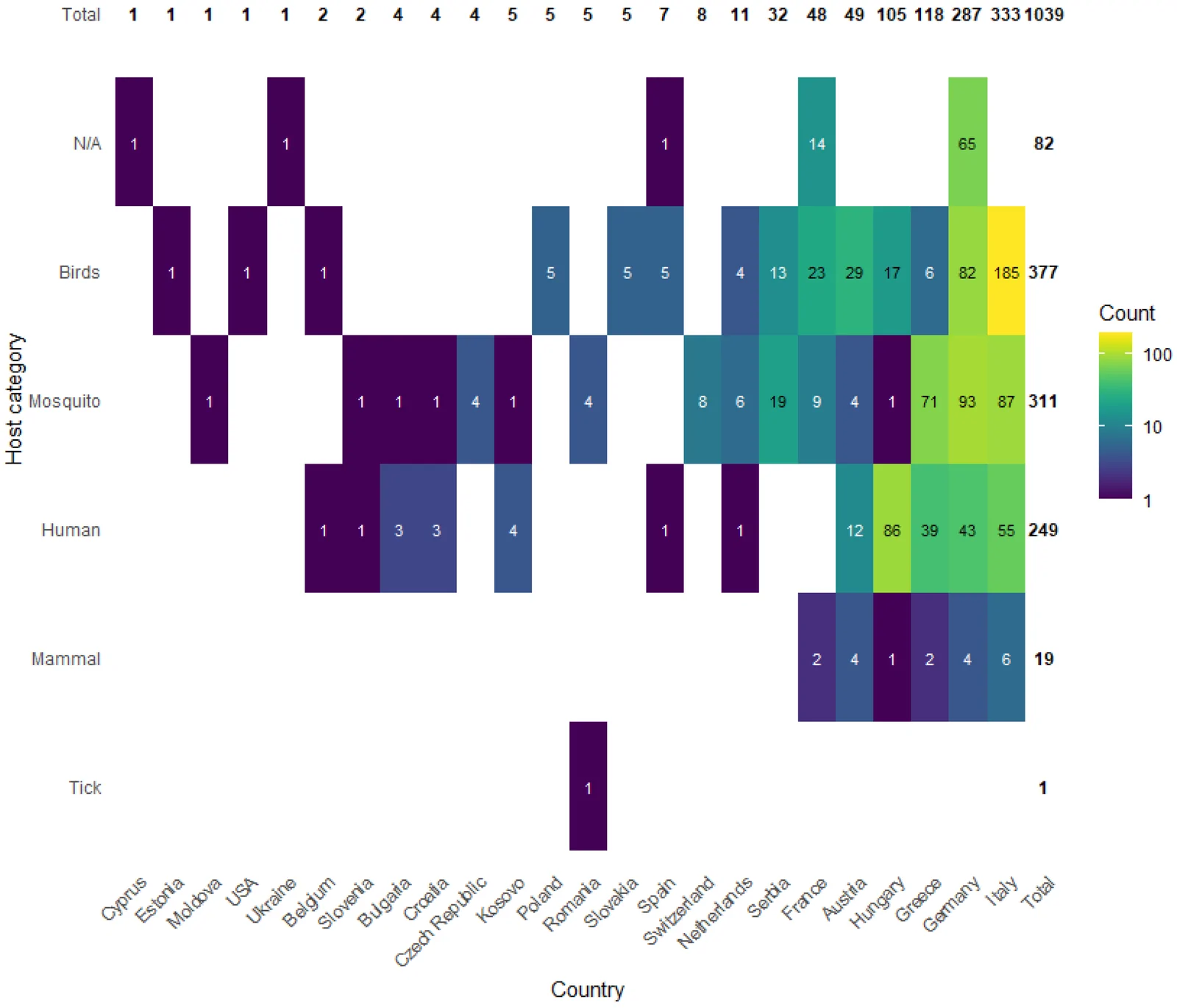
Heatmap indicating the strain counts per country and assigned host category of the created NCBI EU WNV-2 dataset. N/A indicates strains without reported host metadata. USA/WNV1 strain used as outgroup included. Plot created with R 4.4.0.

**Figure 3:**
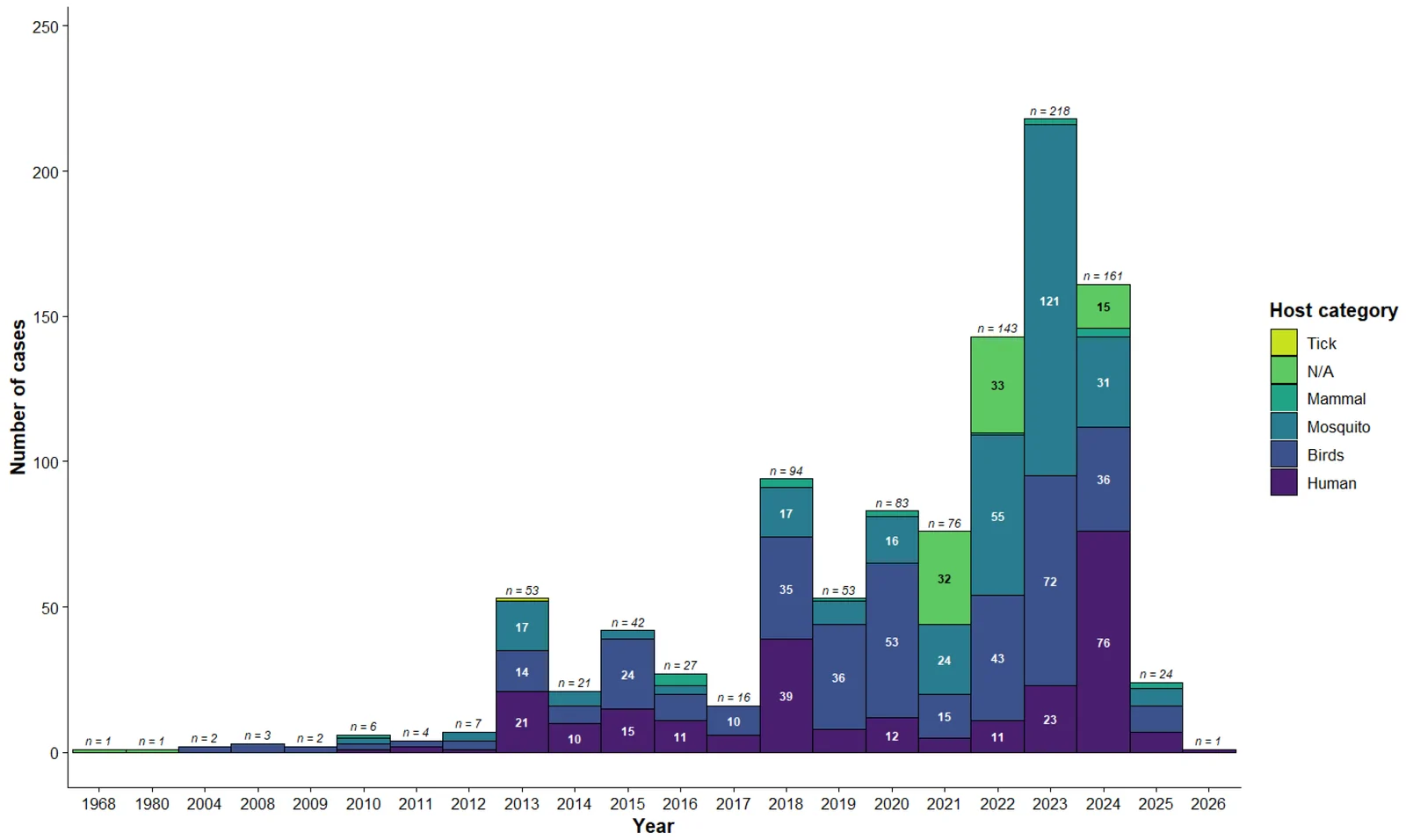
Barplot featuring year and host category of the EU WNV-2 whole-genome sequence dataset, excluding the WNV-1 outgroup sequence. Plot created with R 4.4.0.

For the phylogenetic analysis, a local Nextstrain analysis was implemented according to the Nextstrain documentation (Nextstrain version: 8.5.4, augur version: 28.0.1, Auspice version: 2.65.0, nextclade version: 3.9.1) [31] (json file in Supplementary Materials S1). The used reference sequence was PV637093.1 (HU, 2014). To place the Austrian sequences into a broader European context, 990 additional whole-genome European WNV-2 sequences were added to the Austrian strains from the public NCBI nucleotide database (accession date: 1 July 2026, Supplementary Materials S2). A North American WNV-1 strain (FJ151394.1) was included as an outgroup. The following corresponding metadata were included in the analysis: the sampling date, geographic location (country-level), and host. If no host data were available or no species description was included in the NCBI entry, N/A was indicated. To elevate the analysis, host entries were sorted into the following categories: mammal (excluding humans), human, bird, mosquito and tick. The obtained data distribution of the reported countries and host category is indicated in Figure 2, while the host distribution per year is depicted in Figure 3.

For the bird entries, the *Corvus* species constituted the main proportion of avian host species, with 38.5%, including magpies (*Pica pica*, 13%), hooded crows (*Corvus cornix*, 9%) and unspecified *Corvus* sp. entries dominating. Goshawks (*Astur gentilis*) make up 19.6% of avian host species entries behind the *Corvus* species. Interestingly, only 19 non-human mammalian host species were found in the curated dataset, with the main proportion (*n* = 14) being domestic horses.

The nucleotide substitution rate within the European WNV-2 Nextstrain build was assessed via the molecular clock function within the Nextstrain build. Clade assignment according to previously published nomenclatures was only done on the clade level [3,7,8]. WNV-2 subclade D2 was identified through the previous subclade assignment in the Austrian WNV-2 phylogenetic analysis.

Austria’s geographic location was explored as a potential transmission and clade divergence hotspot via the annotation for each (sub)clade branch provided by the augur tool embedded within the Nextstrain suite [31]. For this analysis, the divergence dates, confidence intervals and geographic state assignments of major nodes were extracted from the ancestral trait reconstruction generated by augur [32].

The geographic spread patterns of the respective clades and subclade D2 were inferred from the discrete ancestral state reconstruction implemented in augur using country metadata. Geotemporal dissemination videos were generated using the temporal and geographic visualization functions implemented in Auspice.

To investigate the phylogenetic structure of the WNV-2 clade E in greater detail, the European Nextstrain phylogeny was filtered to clade E and visualized using branch divergence instead of temporal scaling. Austrian sequences were highlighted to facilitate the identification of country-specific clusters.

## 3. Results

### 3.1. Whole-Genome Sequencing

The reported method for whole-genome amplification and subsequent Illumina paired-end sequencing was applied successfully to all selected Austrian WNV-2 samples collected between the years 2008 and 2025, spanning multiple subclades of the WNV-2 lineage as detailed in Section 3.2.2, thus indicating a reliable primer design for Austrian strains. For 23 samples, a horizontal coverage of 99% was achieved and 93% for a single sample (AGES internal code: 2053/24). Across the study, no generated sequence data yielded a mean depth coverage lower than 100. Samples with a Cq-value < 28 could be successfully sequenced. All generated sequences were made available via the NCBI GenBank, accession numbers PZ698354-PZ698377. The identifier assignment to the respective NCBI entries can be found in Table Supplementary Materials S3 in the Supplementary Materials.

### 3.2. Phylogenetic Analysis and Clade Assignment of Austrian WNV-2 Whole-Genome Sequences

#### 3.2.1. Phylogenetic Tree Evaluation with TreeTime

**Figure 4:**
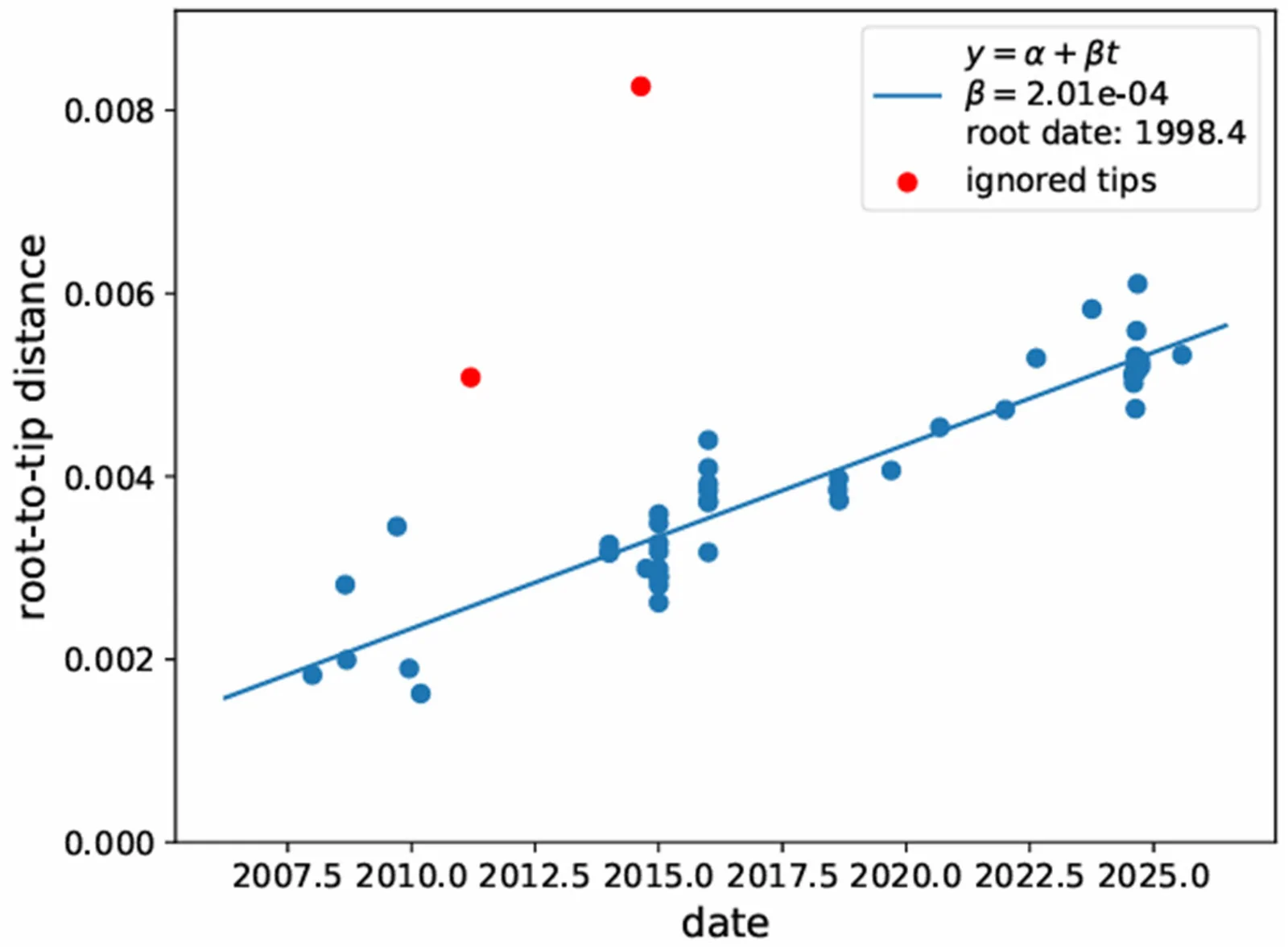
TreeTime root-to-tip regression of the Austrian maximum-likelihood tree created by IQ-TREE.

The evaluation of the created Austrian WNV-2 maximum-likelihood tree with TreeTime revealed a nucleotide substitution rate of 2.01 × 10^−4^ per site and year, with a respective R^2^ of 0.86 and a root date of 1998.40. Two outliers were identified by TreeTime (KP780839 and KP780840), as seen in Figure 4. The best-fitting model according to the MFP algorithm of IQ-TREE was the model TIM2 + F + G4.

#### 3.2.2. Analysis of the Austrian WNV-2 Phylogeny

**Figure 5:**
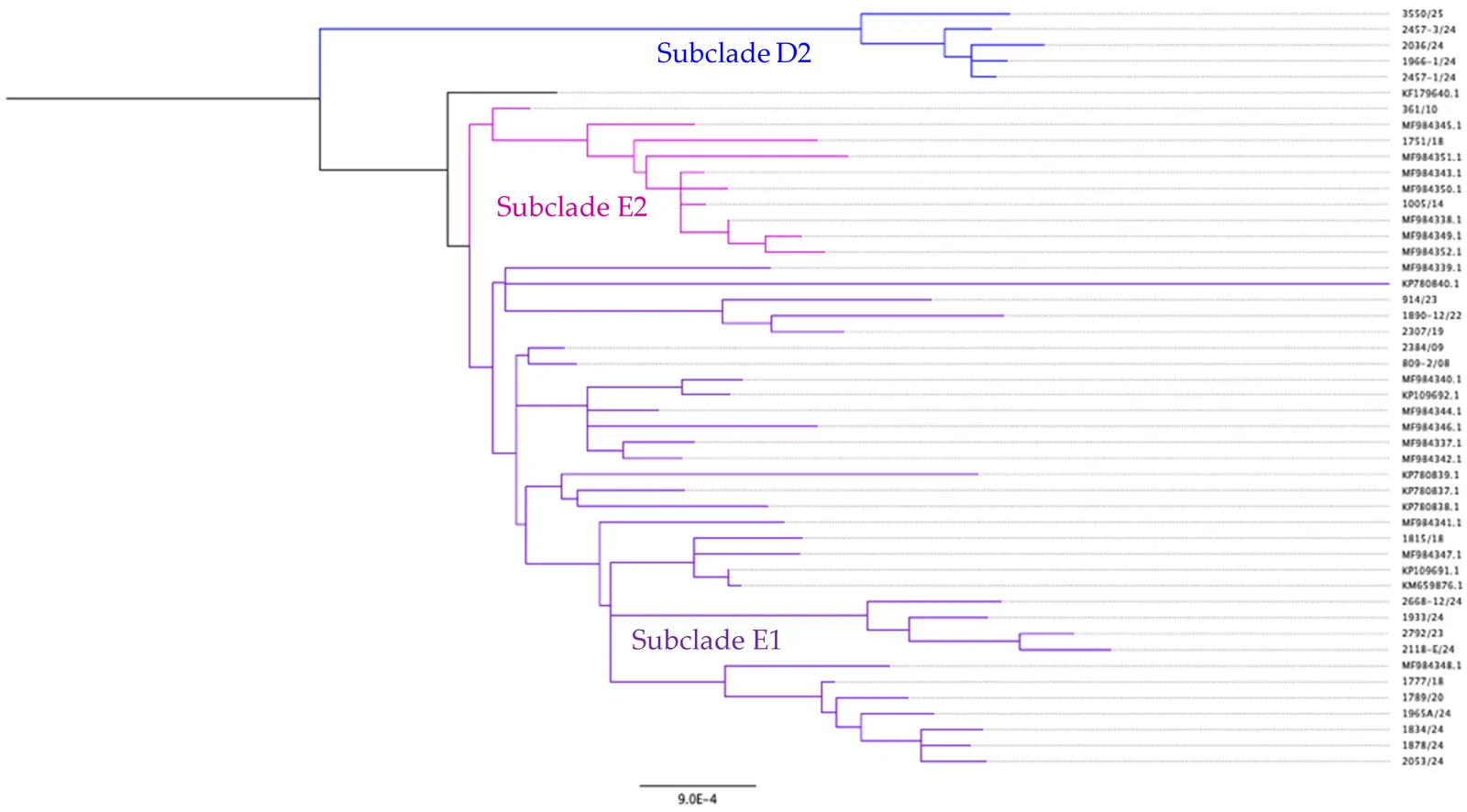
Maximum-likelihood phylogeny of Austrian WNV-2 whole-genome sequences with the respective assigned subclades, visualized with FigTree (version 1.4.5).

The maximum-likelihood analysis revealed the presence of three WNV-2 subclades (subclades D2, E1 and E2) in Austria between 2008 and 2025, as seen in Figure 4. Interestingly, the sample KF179640.1 was flagged by the VSEARCH as subclade E2 with a 99.7% identity, but seemed to cluster outside the defined E2 subclade in the phylogeny and therefore was not indicated as E2 in Figure 5.

**Figure 6:**
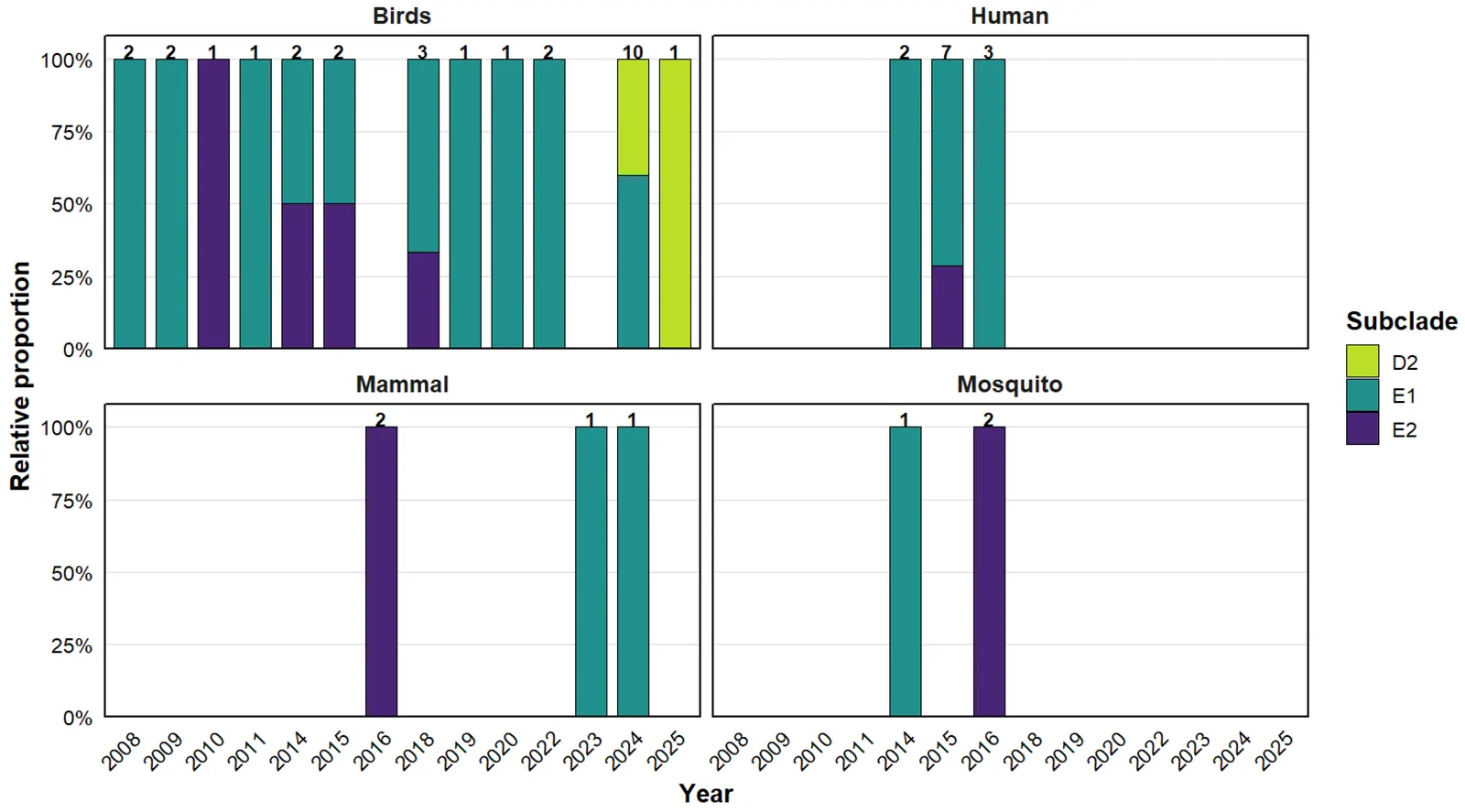
Temporal changes in the distribution of WNV-2 subclades (D2, E1, E2) across different host species. Bars represent 100% of samples per year within each host; numbers denote sample size (*n*). Plot created with R 4.4.0.

Furthermore, these subclade assignments revealed the co-circulation of the WNV-2 subclades E1 and E2 in the years 2010, 2014, 2016 and 2018, as well as the occurrence of the subclades D2 and E1 at the same time in 2024 in Austria. After reviewing the metadata, no distinct host, geographical or time connection can be drawn for either subclade. The presence of subclade D2 in Austria has only been detected in birds to date; however, the further sequencing of future cases might reveal the presence of this subclade in mammals as well. These findings suggest that the respective subclades were co-circulating within the same geographic area and possibly shared the same ecological transmission system. Furthermore, subclade E2 was not detected in any of the analyzed samples in Austria after 2018. The subclade distribution for each host between 2008 and 2025 is depicted in Figure 6.

### 3.3. Phylogenetic and Epidemiological Analysis of Austrian WNV-2 Sequences in the Broader European Context

**Figure 7:**
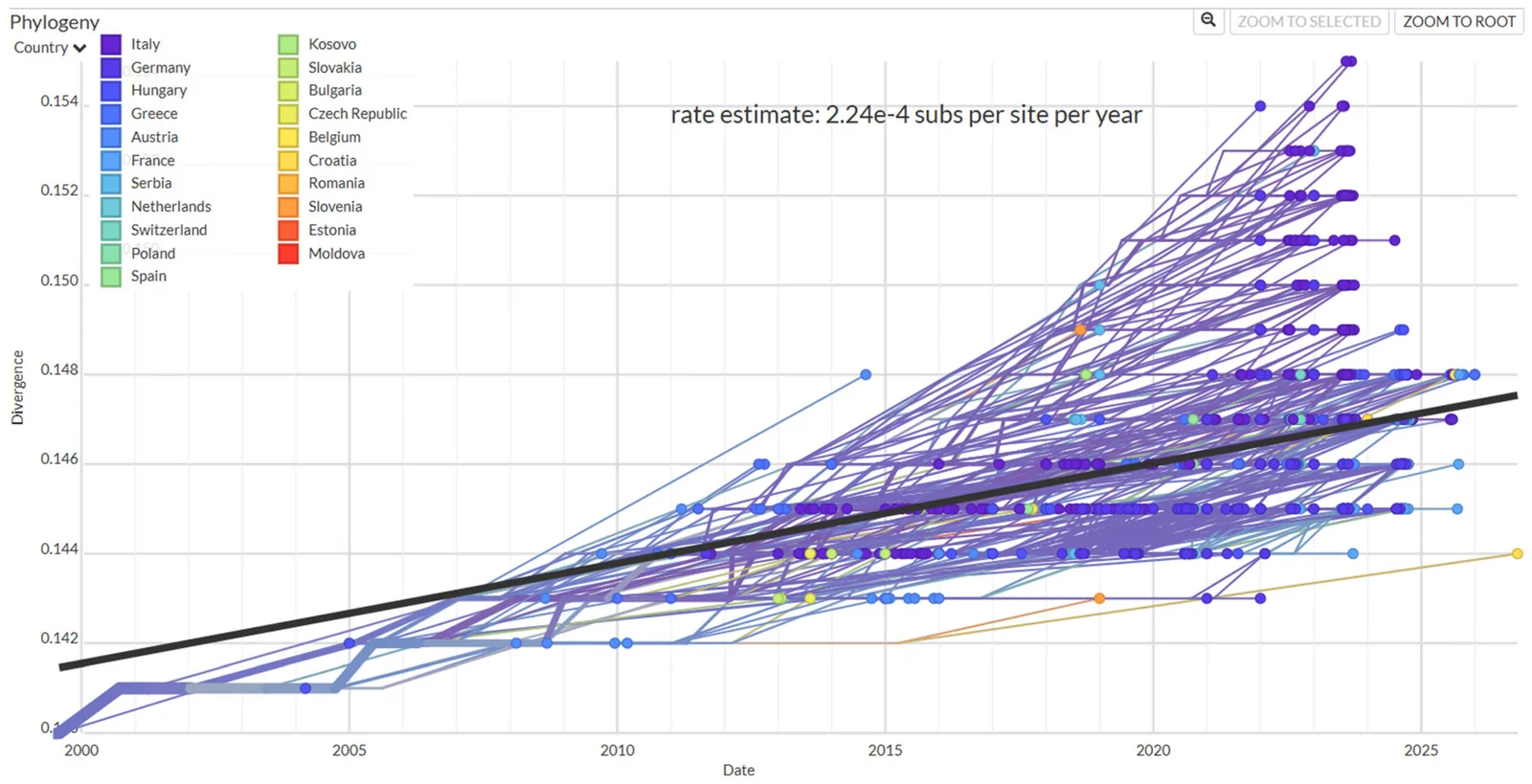
Root-to-tip Nextstrain regression analysis of the molecular clock signal. The root-to-tip divergence is plotted against the sampling date; the regression line indicates the inferred evolutionary rate.

The root-to-tip regression analysis of the European WNV-2 build yielded an estimated evolutionary rate of 2.24 × 10^−4^ substitutions per site per year (SD = 1.25 × 10^−5^) (Figure 7).

**Figure 8:**
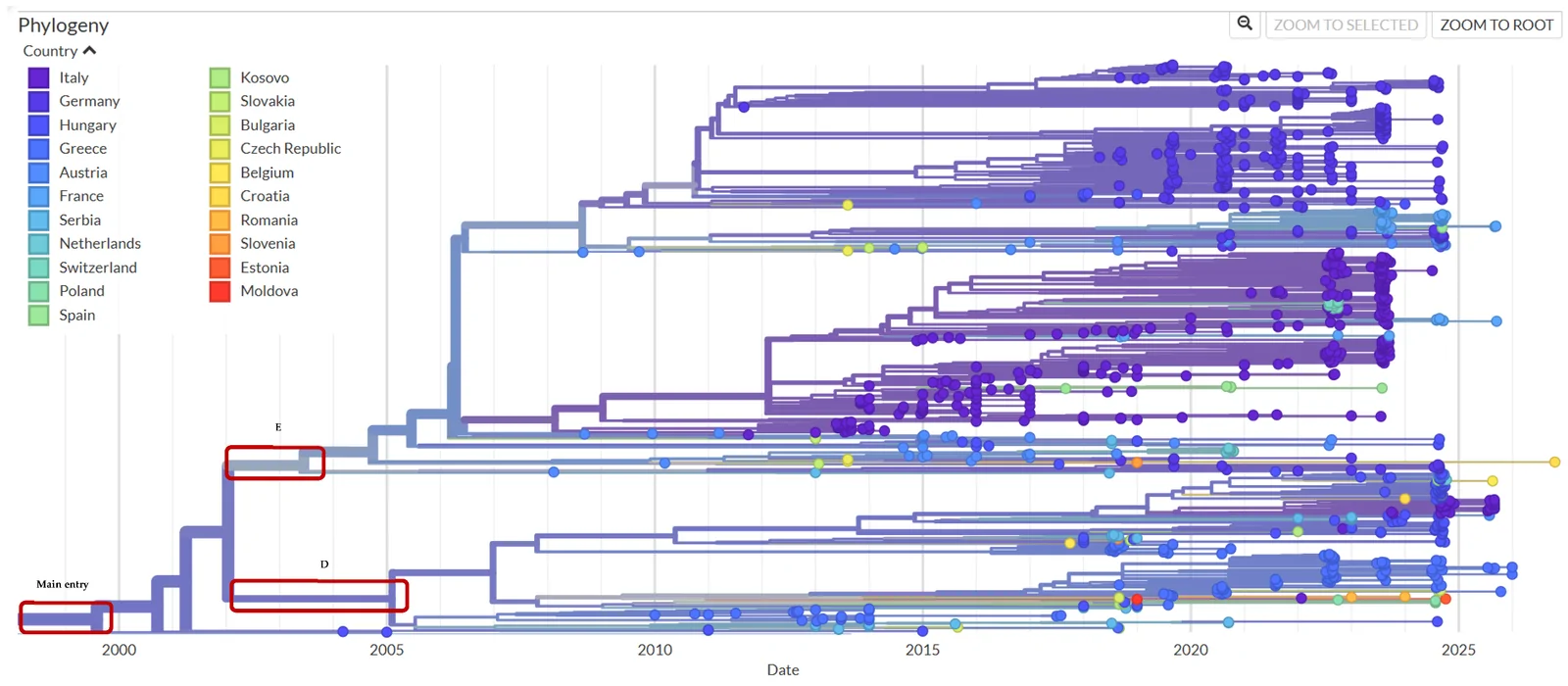
Complete European WNV-2 phylogenetic tree visualized by Nextstrain; the numbers and corresponding red boxes denote the branches defining the emergence of clades D and E.

An investigation of the date and location of divergence of the respective subclades in our dataset implied that the earliest major diversification events of European WNV-2 strains were likely placed within the Hungary/Austria region during the early 2000s. The main entry event was dated to April 2000 and assigned to Hungary, whereas the subsequent split into clade D was inferred for 2005, followed by the emergence of subclades D1–D3 between late 2005 and 2007. Clade E was dated to June 2003 and showed a shared assignment to Austria and Hungary, while the downstream subclades of clade E were predominantly or exclusively associated with Austria. The results indicate that Austria and Hungary represent key geographic locations in the early diversification and regional spread of European WNV-2. However, these reconstructions were influenced by the uneven sequence availability across countries, and therefore, future additional samples might influence the analysis outcome. The geographic and temporal raw data interpreted in this study are shown in Table 4 below, and the corresponding data to the mentioned node tips can be found in Supplementary Materials S1. The complete phylogeny of this data set is shown in Figure 8 with corresponding notation of the clades D and E.

#### 3.3.1. Phylogenetic and Epidemiological Analysis of Austrian WNV-2 Sequences of Subclade D2 Strains in the Broader European Context

**Figure 9:**
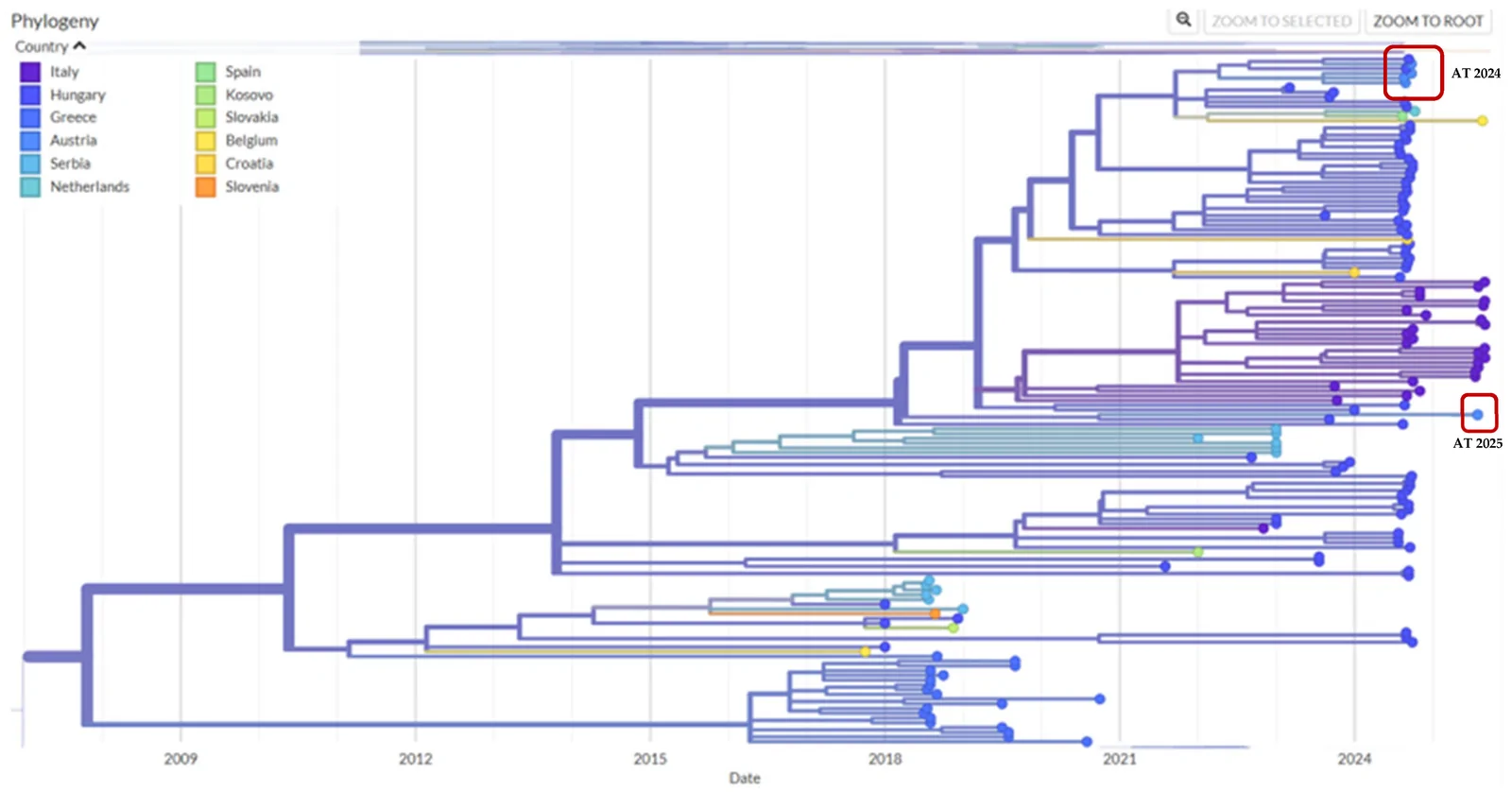
Enlarged section of the European WNV-2 phylogeny showing subclade D2, visualized in Nextstrain. Austrian sequences from 2024 form a distinct cluster within D2, while the Austrian sequence from 2025 is phylogenetically separated from this cluster, indicating a likely independent virus introduction event. The interpretation of regional dissemination routes remains limited by the uneven whole-genome sequence availability across neighboring countries.

The detailed phylogenetic analysis of subclade D2, which contains the entirety of all Austrian WNV-2 strains belonging to clade D, revealed the presence of two clearly separated Austrian D2 clusters (Figure 9).

**Figure 10:**
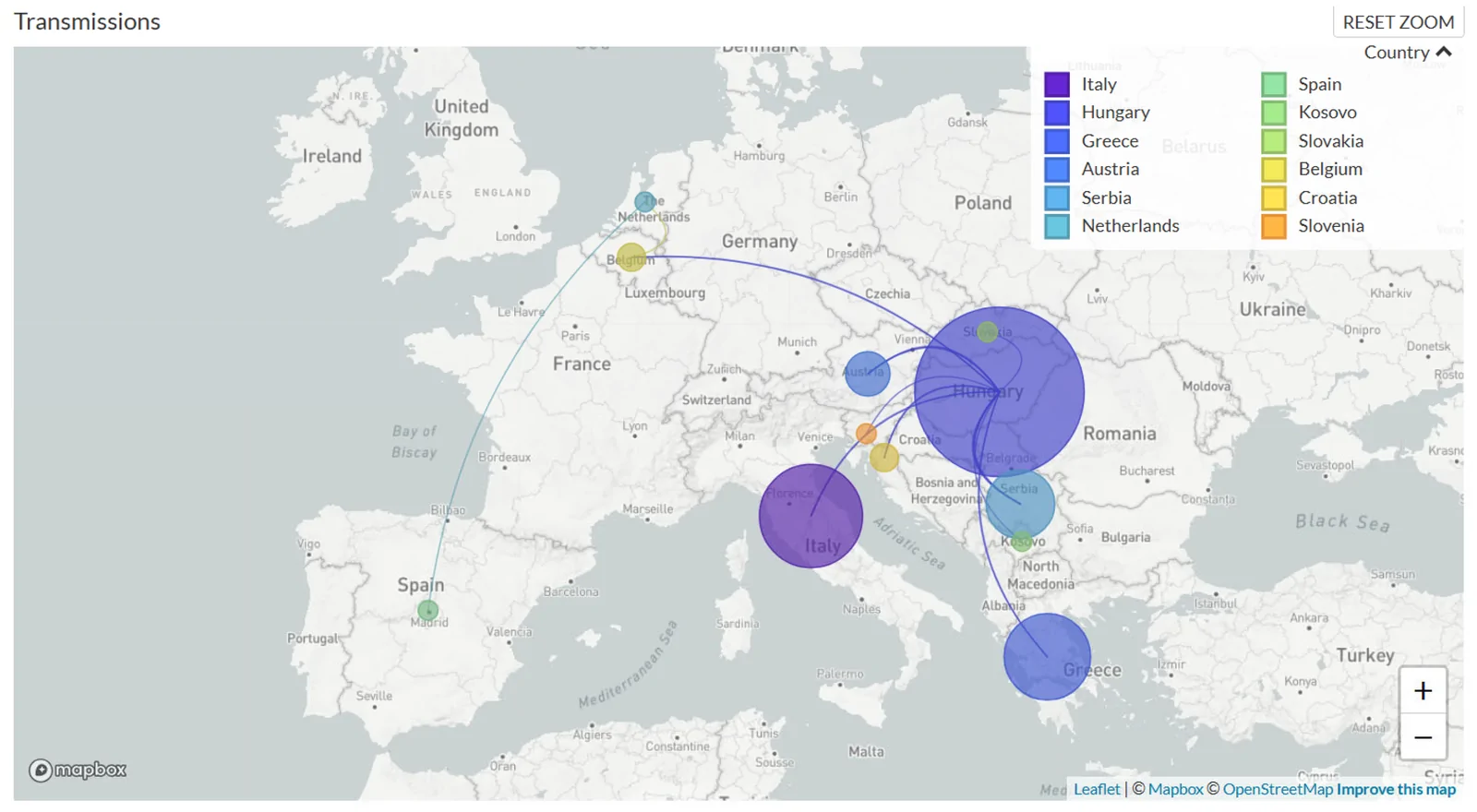
Nextstrain-based reconstruction of inferred WNV-2 dissemination events for subclade D2 across Europe. Hungary occupies a central position within the reconstructed transmission network, although the exact dissemination routes should be interpreted cautiously due to the uneven sequence availability among European countries (represented by the circle size). The circle locations indicate countries only and do not represent precise sampling locations.

The Austrian sequences obtained in 2024 form a cluster together with Hungarian D2 sequences sampled in 2024, suggesting a common epidemiological background. In contrast, the Austrian sequence detected in 2025 did not group with the Austrian 2024 cluster, but instead clustered together with Hungarian and Italian D2 strains collected in 2025. This phylogenetic separation indicates that the Austrian 2025 strain represents a distinct introduction event rather than the continued circulation of the D2 variants detected in Austria during 2024. Although the precise transmission pathway remains uncertain as the data availability for 2025 WNV-2 strains is, at the present point, limited, the observed clustering pattern is consistent with two independent introductions of D2 into Austria within a short time period. The Nextstrain geotemporal spread model, based on genomic data (Figure 10, as well as video Supplementary Materials S4), inferred an initial spread of clade D2 from Hungary towards south-eastern Europe, with predicted early transmission events into Slovenia, Serbia, and Greece, as well as an independent introduction into Belgium, where the introduction marked the first recorded case of WNV detected via wild bird surveillance (NCBI accession number: PX906586.1) [33]. The subsequent spread was reconstructed towards Slovakia and Italy, whereas introductions into Austria and Kosovo were implied comparatively late in the inferred transmission history. Overall, the phylogeographic pattern suggests two major dissemination routes originating from Hungary: an early southward expansion along the Balkan Peninsula and a later westward spread towards Italy and Austria. This finding might be changed by further publicly available sequences provided by other European countries. The clade D2 dataset included two publicly available sequences from 2024, PQ462527.1 from the Netherlands with the NCBI entry featuring Spain as the geolocation and PQ483500.1 from the Netherlands. The latter was obtained from a human infection in a traveler returning from Spain. This recorded travel history represents only circumstantial evidence of a possible epidemiological association with Spain and cannot establish a certain location of infection or a specific origin of the sequence. Therefore, due to the lack of conclusive evidence and the high uncertainty regarding the geographic origin of the Dutch case, the inferred connection shown in Figure 10 should not be considered a well-supported transmission link.

#### 3.3.2. Phylogenetic and Epidemiological Analysis of Austrian WNV-2 Sequences of Clade E Strains in the Broader European Context

**Figure 11:**
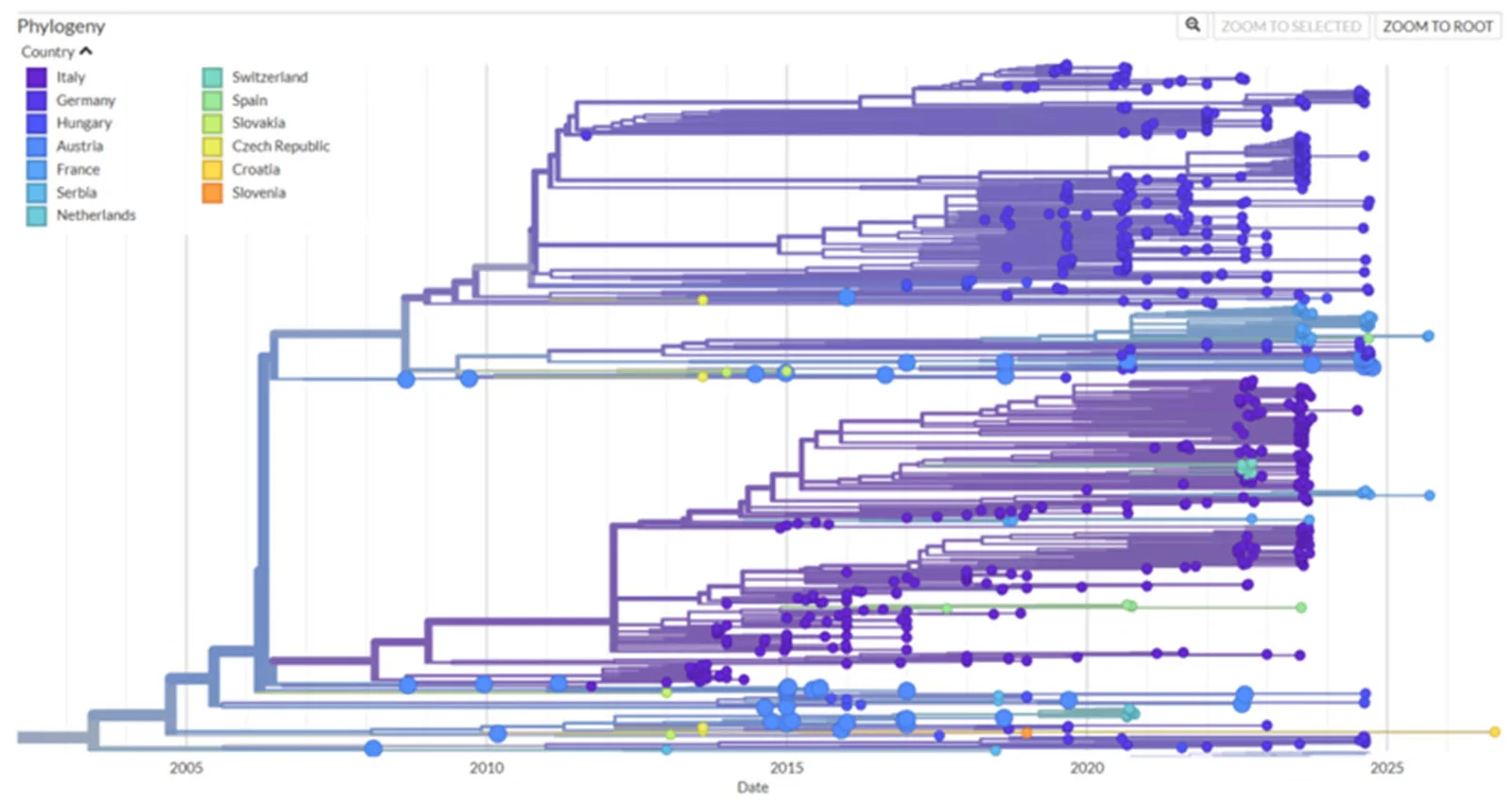
Section of the European WNV-2 phylogeny showing clade E, visualized via Nextstrain. Austrian tips are illustrated by larger circles on the phylogeny tips.

**Figure 12:**
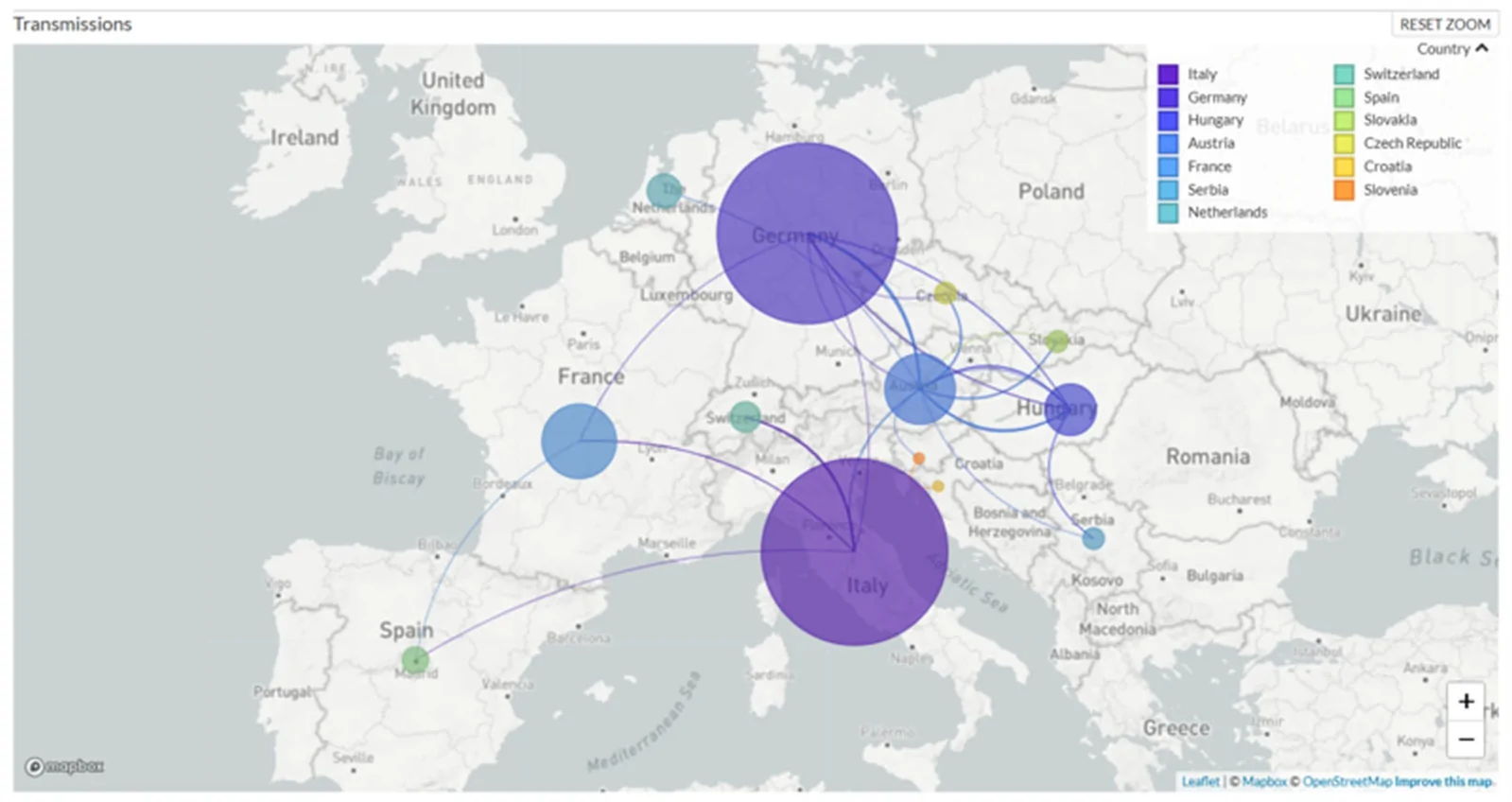
Nextstrain-based reconstruction of inferred WNV-2 dissemination events for clade E across Europe. The circle size represents the number of samples from each country, while the circle locations are shown at the country level. The position of each circle is intended to indicate the corresponding country only and does not reflect the actual geographic location of samples within that country.

In contrast to clade D, clade E exhibited a considerably more complex phylogenetic structure (Figure 11). The phylogeny was characterized by several geographically structured clusters dominated by sequences from individual countries. Supplementary Materials S5, as well as Figure 12, visualizes the derived geographic expansion of Clade E across Europe from its presumed point of emergence in Hungary. The model infers an initial spread to Austria and subsequently to Italy, Germany, and Slovakia, followed by later dissemination into the Czech Republic, Serbia, Slovenia, and Croatia. Further westward expansion was indicated from Italy into France, Switzerland, and Spain, and from Germany into France and the Netherlands. While the overall pattern was consistent with an east-to-west spread, occasional introduction events in the opposite direction were detected between Hungary and Austria and between Austria and Germany.

**Figure 13:**
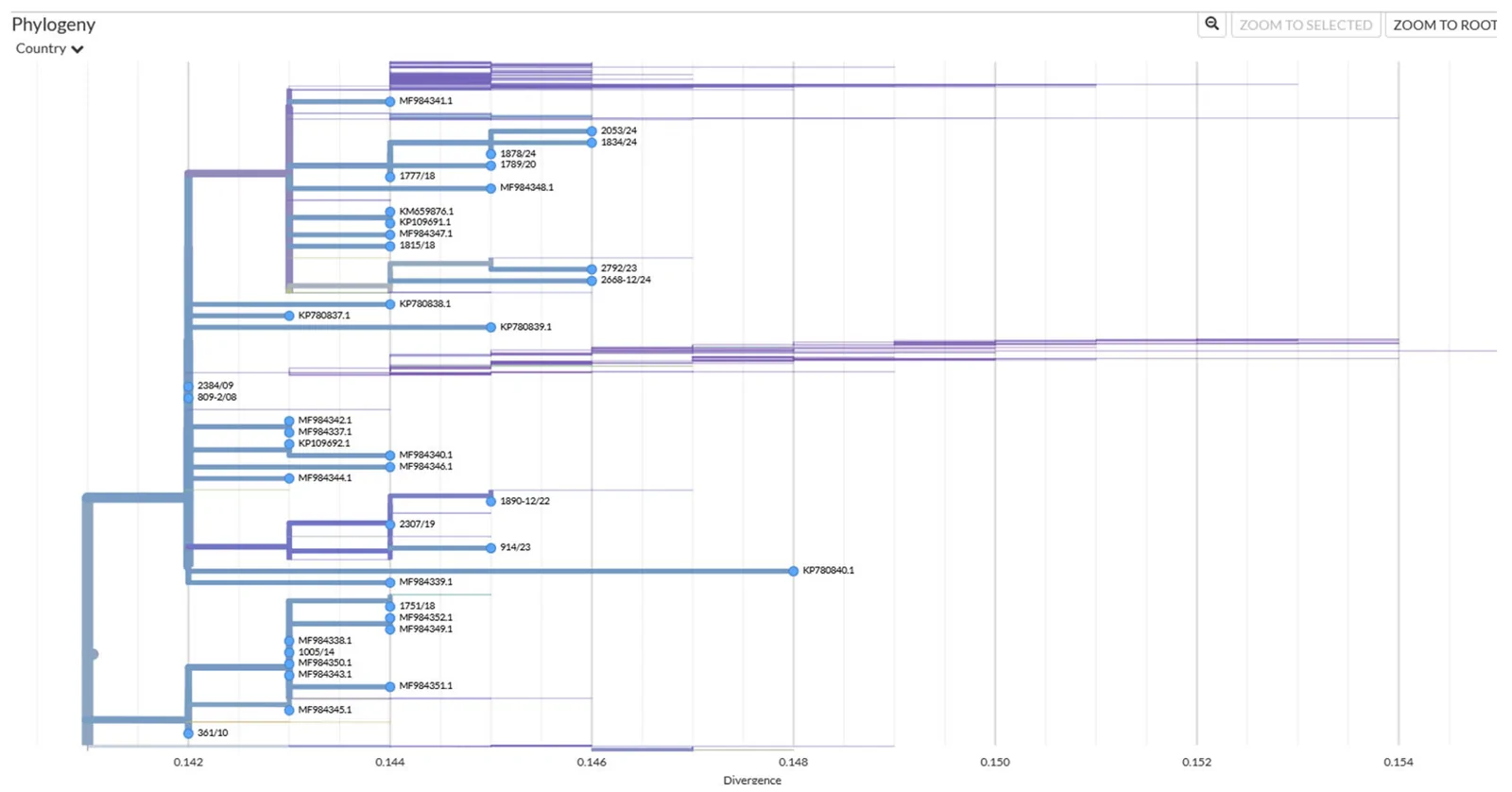
Enlarged divergence-scaled section of the European WNV-2 Nextstrain phylogeny showing clade E. The x-axis represents genetic divergence, thereby emphasizing sequence relatedness by branch length differences within clade E. The view was filtered to highlight Austrian sequences.

The divergence-based phylogeny of clade E (Figure 13) largely recapitulates the Austrian subclade structure previously identified in the Austrian-only phylogeny (Section 3.2.2). Notably, the cluster corresponding to the inferred Austrian subclade E2 contained phylogenetically related entries after 2018 such as strains from Germany 2019 (LR743448, LR743431), the Netherlands 2020 (OP762592–OP762597), and Germany 2022 (PQ119459). The most recent sequence associated with this cluster originated from Croatia in 2026 (PQ738244). Overall, the cluster comprised sequences detected in Austria, Czechia, Slovenia, Croatia, Germany, and the Netherlands. The presence of genetically related strains over a period spanning at least 2008–2026 indicates the possibly continued circulation of this lineage.

## 4. Discussion

The WNV-2 whole-genome sequence data generated in this study closes an important gap in Austrian as well as European WNV-2 epidemiological data, as Austria appears to play an important geographical role in WNV-2 spread. This dataset comprises over 32% of all confirmed Austrian WNV-2 cases detected in animal hosts between 2008 and 2025, covering the period from the virus’s first identification in birds in Austria to the most recent surveillance year, as of conducting this study. These include an equine case and the first European alpaca case of WNV-2 for which a whole-genome sequence is publicly available in the NCBI database; associated sample metadata are provided in Supplementary Materials S2.

The established whole-genome amplification method proved to be reliable for Austrian strains spanning 17 years, as well as multiple subclades, and provides a fast and simple workflow for the wet-lab part. The highest Cq-value of a successfully sequenced sample was 26.9. As the created primer set yields 1500 bp long amplicons, the amplicons can also be sequenced using the Rapid Barcoding Kit by Oxford Nanopore Technologies (Oxford, UK) (ONT) (not tested in this study), depending on the sequencing platform availability for maximal flexibility [29].

The presented Austrian dataset shows the presence of three different WNV-2 subclades in the time span from 2008 to 2025: subclade D2, E1 and E2. Subclade D2 was introduced recently in Austria in 2024. Notably, the D2 strain from 2025 seems to be a new introduction rather than a reintroduction of the strain variants of D2, which were detected in Austria in 2024. The continued monitoring of the detected WNV-2 strains in Austria through whole-genome sequencing is therefore crucial for identifying new entries and their possible entry routes.

The spread of the subclade D2 is inferred to originate from Hungary and subsequent expanding to the neighboring countries. In line with this, the European Nextstrain reconstruction summarized in Table 4 places the inferred divergence of subclade D2 in Hungary around September 2007. Furthermore, the phylogenetic analysis supports the interpretation that recent Austrian D2 detections most likely reflect repeated regional introductions from an already established south-eastern/central European transmission network rather than long-term circulation within Austria.

However, due to sampling bias, the true circulation area and geographical dissemination route of D2 remain unclear. This limitation is particularly relevant because Hungarian sequences constitute more than 10% of all available European entries, whereas several neighboring countries currently lack publicly available WNV-2 whole-genome sequences. The isolated Austrian case detected in 2025, which showed no immediate phylogenetic connection to other European sequences from that year, further supports this concern and highlights the need for broader regional sequencing coverage. Moreover, the observation that two temporally adjacent Austrian D2 detections belonged to different phylogenetic clusters demonstrates the need for an adequate sample size per year to reflect the diversity of WNV-2 introductions.

Furthermore, in 2024, the co-circulation of subclades D2 and E1 could be identified in Austria, while in the years of 2010, 2014, 2016 and 2018, the co-circulation of subclades E1 and E2 was found. Interestingly, while subclade E2 has not been detected in Austria since 2018, the identification of a closely related WNV-2 strain from Croatia in 2026 indicates that this lineage continues to circulate within Europe. The repeated observation of co-circulating lineages highlights the complexity of WNV-2 transmission dynamics and underlines the importance of sustained whole-genome surveillance.

The European WNV-2 Nextstrain build further points to the role of Austria and Hungary as key geographic hubs in the divergence and spread of European WNV-2 lineages, consistent with previous observations [31]. The inferred Hungarian origin of clade D and its major subclades further support the importance of the region in shaping WNV-2 diversity in Europe. Several major diversification events within clade E were inferred to originate either in Austria or at the Austrian–Hungarian region, highlighting this area as a likely long-term hotspot of WNV-2 evolution and diversification in Europe. Nevertheless, these reconstructions should be interpreted with caution due to sampling biases and the limited availability of whole-genome sequences from several neighboring countries. Further sampling and the sharing of whole-genome sequencing data among the European countries is needed to further the understanding of the continued transmission dynamics of WNV-2 in Europe [10].

## Figures and Tables

**Table 1:** Sample identifiers, matrices, host species, respective WNV real-time RT-PCR Cq-values and sampling dates of the samples included in this study.

Identifier	Matrix	Host	Cq-Value	Sampling Date
809-2/08	pooled organs	goshawk	N/A ^1^	9 September 2008
2384/09	pooled organs	gyrfalcon	N/A ^1^	15 December 2009
361/10	pooled organs	goshawk	N/A ^1^	10 March 2010
1010/14	pooled organs	goshawk	N/A ^1^	30 September 2014
1751/18	pooled organs	crow	15.00	14 August 2018
1777/18	pooled organs	goshawk	15.80	20 August 2018
1815/18	pooled organs	goshawk	13.00	23 August 2018
2307/19	pooled organs	goshawk	21.00	12 September 2019
1789/20	pooled organs	European goldfinch	14.00	5 September 2020
1890-12/22	spleen	eastern imperial eagle	24.80	18 August 2022
914/23	pooled organs	bearded vulture	24.70	XX August 2022 ^2^
2792/23	pooled organs	alpaca	25.60	2 October 2023
1834/24	pooled organs	magpie	15.54	31 July 2024
1878/24	pooled organs	crow	17.40	6 August 2024
1933/24	brain	gyrfalcon	19.38	13 August 2024
1965A/24	pooled organs	goshawk	12.50	19 August 2024
1966-1/24	pooled organs	crow	23.76	19 August 2024
2036/24	pooled organs	crow	12.61	26 August 2024
2053/24	pooled organs	crow	10.99	28 August 2024
2118-E/24	brain	horse	26.87	4 September 2024
2457-1/24	pooled organs	raven	24.64	23 September 2024
2457-3/24	pooled organs	crow	13.60	23 September 2024
2668-12/24	pooled organs	sparrow	11.70	3 October 2024
3550/25	pooled organs	carrion crow	23.00	29 July 2025

^1^ Analyzed via two-step heminested RT-PCR. ^2^ No exact sampling date available.

**Table 2:** Designed primer sets for WNV-2 with primalscheme.

**Primer Set PCR1**	**Sequence 5′–3′**
WNV2_1500_100_1_LEFT	GATGTCTAAGAAACCAGGAGGGC
WNV2_1500_100_1_RIGHT	TGTCTATTCCTGACCGTGGCT
WNV2_1500_100_3_LEFT	CAGATAAACCACCACTGGCACA
WNV2_1500_100_3_RIGHT	ACTAGGACTAGGAGTGCAAGCA
WNV2_1500_100_5_LEFT	ATACCACCACTGGCGTTTACAG
WNV2_1500_100_5_RIGHT	TATTCCCCGTCCATGGTGTACA
WNV2_1500_100_7_LEFT	TTGTGGACGTTGGTGTGTCAG
WNV2_1500_100_7_RIGHT	GGTGATAGTGTCCCATGGTTTTGA
WNV2_1500_100_9_LEFT	CGAAGGTTCTTGAACTGCTGGA
WNV2_1500_100_9_RIGHT	TTACAGCTTCAGCCAAGTTGGG
**Primer Set PCR2**	
WNV2_1500_100_2_LEFT	AGTAACAGAGACTTCCTGGAGGG
WNV2_1500_100_2_RIGHT	AACTCCTCCAACCGCAAGAAAC
WNV2_1500_100_4_LEFT	AGAGTGGGAAGCTCATCACAGA
WNV2_1500_100_4_RIGHT	GGCTCCACGACAATCATTTGGA
WNV2_1500_100_6_LEFT	TGAGGAAGGTGATGGAAGAGTCA
WNV2_1500_100_6_RIGHT	TCTGCATGACTGGAGTTGTTCG
WNV2_1500_100_8_LEFT	AAGAAAACATGGAAGGGACCCC
WNV2_1500_100_8_RIGHT	ATCAACTCGTCTTGTCCACGAC

**Table 3:** NCBI references used for subclade assignment.

WNV-2 Clade	NCBI Reference
D1	MN481593
D1	KX375812
D2	MW751834
D2	OL840882
D3	OP804520
D3	OQ053522
E1	MH244510
E2	MF984351

**Table 4:** Tabular overview of previously named WNV-2 subclades, including inferred country of divergence, inferred divergence date, divergence date confidence interval, Nextstrain build node name and number of terminal tips.

(Sub)Clade	Country	Inferred Date	Date Confidence Interval	Node Name	No. of Terminal Tips
Main entry event	Hungary (100%)	13 April 2000	25 May 1998–16 June 2001	0000287	1020
D	Hungary (100%)	4 February 2005	16 March 2004–21 May 2006	0000244	282
D1	Hungary (100%)	9 December 2005	8 October 2004–27 June 2006	0000245	39
D2	Hungary (99%)	18 September 2007	15 August 2006–28 February 2009	0000128	145
D3	Hungary (99%)	14 August 2007	18 August 2006–4 January 2009	0000147	98
E	Austria (60%)/Hungary (40%)	15 June 2003	17 July 2002–21 March 2006	0000296	735
E1	Austria (93%)/Germany (7%)	24 August 2008	10 May 2007–30 August 2009	0000698	336
E2	Austria (100%)	31 January 2008	3 September 2006–24 September 2009	0000315	28

## Data Availability

The original data presented in the study are openly available in the NCBI Genbank, accession numbers PZ698354-PZ698377.

## Supplementary Materials

The following supporting information can be downloaded at: https://www.mdpi.com/article/10.3390/pathogens15101061/s1: Nextstrain Auspice json file Supplementary Materials S1: SUP1_WNV-2_EU_nextstrainbuild_auspice.json; WNV-2 EU metadata table Supplementary Materials S2: SUP2_WNV-2_EU_nextstrain_metadata.tsv; AGES-NCBI WNV-2 accession number table Supplementary Materials S3: SUP3_AGES-NCBI_WNV-2 accession_numbers.xlsx; video geotemporal transmission across Europe of subclade D2 Supplementary Materials S4: SUP4_cladeD2_geotemporal_WNV-2.mp4.; video geotemporal transmission across Europe of clade E Supplementary Materials S5: SUP5_CladeE_geotemporal_WNV-2.mp4.

## Abbreviations

The following abbreviations are used in this manuscript:

AGES	Austrian Agency for Health and Food Safety
bp	base pair
CEC	central European clade
CI	confidence interval
Cq	cycle threshold
EU	European Union
GC	guanine–cytosine
HERA	Health Emergency Preparedness and Response Authority
IVET	Institute for Veterinary Disease Control
MFP	ModelFinder Plus
ML	maximum likelihood
N/A	not available
NCBI	National Center for Biotechnology Information
NGS	next-generation sequencing
ONT	Oxford Nanopore Technologies
PCR	polymerase chain reaction
RT-PCR	reverse-transcription polymerase chain reaction
SD	standard deviation
SEEC	south-eastern European clade
WGA	whole-genome amplification
WGS	whole-genome sequencing
WNV	West Nile virus
